# Evaluation of corn-fermented protein as a dietary ingredient in extruded dog and cat diets

**DOI:** 10.1093/tas/txad032

**Published:** 2023-03-21

**Authors:** Spencer C Smith, Charles G Aldrich

**Affiliations:** Department of Grain Science, Kansas State University, Manhattan, KS 66506, USA; Department of Grain Science, Kansas State University, Manhattan, KS 66506, USA

**Keywords:** corn-fermented protein, digestibility, distillers dried grains, extrusion, palatability, pet food

## Abstract

Most pet foods utilize traditional ingredients like corn, wheat, and soy. These ingredients and other grains, such as distillers dried grains (DDG), have been used by the pet food industry. Corn-fermented protein (CFP) is a nutrient-dense enhancement on DDG but has not been evaluated in pet food. Therefore, it was the objective of this study to determine the effect of CFP in the production of extruded pet diets, and to determine the effect on nutrient utilization (digestibility) and stool consistency in dogs, and palatability in dogs and cats. Experimental diets with treatment protein sources (corn gluten meal [CGM], soybean meal [SBM], and CFP) were produced in triplicate using a single-screw extruder. Processing parameters and kibble samples were collected at timed intervals during diet production. Kibbles were evaluated for physical dimension and texture. No differences (*P* > 0.05) were observed in any physical dimension or texture parameters evaluated, with exception of radial expansion, which was lower (*P* < 0.05) for CFP kibble compared to others. The CFP kibble required a smaller (*P* < 0.05) mass restriction valve opening, to keep similar bulk density among dietary treatments. However, there was no difference (*P* > 0.05) in specific mechanical energy among treatments during diet production. Twelve beagles were fed the experimental diets in a 3 × 3 replicated Latin Square design in which four dogs were randomly assigned to each of three treatments for each period. Diets were formulated to be isonitrogenous and were supplemented with titanium dioxide to serve as an external marker in order to estimate apparent total tract digestibility. Dogs were housed individually and fed twice daily, and water was available ad libitum. Feces were collected after feedings. The diet produced with CGM was more digestible (*P* < 0.05) than CFP and SBM for dry matter, organic matter, crude protein, crude fat, and gross energy. Further, the CFP diet was also less (*P* < 0.05) digestible than the SBM diet for dry matter and organic matter. Dogs fed the diet containing CFP had higher (*P* < 0.05) fecal mass than those fed SBM and CGM. The CFP diet also resulted in a higher fecal score (*P* < 0.05) than those fed diets with the CGM diet, but similar (*P* > 0.05) to the SBM diet. For palatability assessment, dogs had a preference (*P* < 0.05) for CGM over SBM or CFP, but cats showed a preference (*P* < 0.05) for SBM and CFP over CGM. Results indicate that CFP is acceptable for use in dog and cat diets. Further research should be conducted to evaluate the use of these ingredients at lower inclusion levels.

## INTRODUCTION

The pet food market is rapidly growing and currently contributes approximately 40% to total sales in the pet industry, more than any other factor (APPA, 2021). While there are many new pet food formats being introduced to the market (e.g., freeze-dried, raw, etc.), dry foods consistently dominate the volume (Pet Food Industry, 2021). Humanization has been a major influence on market trends in the pet food industry. Today’s “pet parents” want their animals to eat as well as they do and are beginning to seek options in ingredient composition that reflect their own purchases (Boya et al., 2015). According to Pet Food Industry magazine (2015), 55% of pet owners are concerned about the amount of “fillers,” such as grains and meat byproducts, in their pet’s diets. However, despite these trends, 81% of dog foods and 85% of cat foods still utilize traditional ingredients like corn, wheat, and soy (Packaged Facts, 2016). Use of plant-based ingredients and coproducts may prove economically beneficial in this rapidly growing market (Silva et al., 2016). While less nutrient-dense than animal proteins, it has been found that there is less variation in nutritional content between plant proteins relative to animal protein meals (Clapper et al., 2001). Protein concentrates from corn and soy have been used successfully in pet foods for decades. Improvements in processing technology and coproducts from distilling grains from ethanol production have created new variations in these base proteins. Therefore, new alternatives such as corn-fermented protein (CFP) should be considered.

Traditional distillers dried grains with solubles (DDGS) have been utilized by the livestock industry for decades due to their high levels of protein, fat, and fiber (Lodge et al., 1997; Batal and Dale, 2006). As a coproduct of ethanol production, the ingredient is readily available and cost-effective (de Godoy et al., 2009). Additionally, the ingredient is very sustainable. The pet food industry has long been practicing sustainability by utilizing coproducts from plant-derived protein sources, such as soybean meal (SBM), or corn gluten meal (CGM; Alonzo, 2017). While these common coproducts have been utilized by the industry for decades, it would be relevant to look for similar alternatives, such as grain coproducts from distillation. CFP is produced using post-fermentation separation technologies to split protein and yeast from fiber prior to drying. This process utilizes a series of screens and centrifuges to separate approximately 20% of total DDGS volume into the new high-protein ingredient, while the remaining 80% is sold as DDGS. These CFP contains twice as much protein as traditional DDGS (Belyea et al., 2004) and may be a viable option for inclusion in pet foods as a protein concentrate (Table 1). An evaluation of the ingredient regarding its effects on extrusion processing, nutrient utilization, and animal acceptance will provide valuable details about its utility. Therefore, it was the objective of this study to determine the effect of CFP in the production of extruded pet diets, and to determine the effect on nutrient utilization (digestibility) and stool consistency in dogs, and palatability in dogs and cats. It was hypothesized that it would be possible to create a kibble of similar size, shape, and texture among treatments using CFP, with minimal changes to processing parameters, and that the acceptability and digestibility of the CFP diet would be similar to the CGM and SBM diets.

**Table 1.** Analyzed nutrient composition of experimental ingredients, expressed on a dry matter basis

	Experimental ingredients^*^		
Item, %	CGM	SBM	CFP
Moisture	10.17	12.33	6.71
Crude protein	74.70	54.50	54.40
Crude fat	1.76	1.28	4.18
Total dietary fiber	4.24	15.22	28.08
Ash	1.11	6.96	4.57
Starch	13.87	4.00	2.73

^*^Corn gluten meal (CGM), soybean meal (SBM), and corn-fermented protein (CFP).

## MATERIALS AND METHODS

All animal use was approved by the Kansas State University Institutional Animal Care and Use Committee (IACUC) prior to the beginning of the study and complied with the National Institutes for Health guide for the care and use of Laboratory animals (AUP 3883; NIH Publications No. 8023, revised 1978).

### Diet Formulation

Diets with three different plant protein sources (CGM [Fairview Mills, Seneca, KS], SBM [Fairview Mills, Seneca, KS], and next generation-distillers dried grain [CFP; POET Bioproducts, Sioux Falls, SD]) were formulated to be to contain equal amounts of protein from test ingredients, with remaining mass made up of corn starch (Tables 2). Diets were formulated to meet nutritional requirements for adult maintenance for both dogs and cats. Chromium sesquioxide (2.5 g/kg) and titanium dioxide (4.0 g/kg) were added to serve as external markers to estimate fecal output in order to compute apparent total tract nutrient digestibility (ATTD); however, for this paper, only the titanium results will be discussed.

**Table 2.** Diet composition and nutrient analysis for experimental treatments

	Diet^*^		
Ingredient, %	CGM	SBM	CFP
Corn	33.9	33.9	33.9
Chicken meal, low ash	28.9	28.9	28.9
CGM	20.5	—	—
SBM	—	24.8	—
CFP	—	—	25.0
Corn starch	4.5	0.3	—
Beet pulp	4.0	4.0	4.0
Vitamins and minerals	1.35	1.35	1.35
Titanium dioxide	0.4	0.4	0.4
Chromium sesquioxide	0.25	0.25	0.25
Fish oil	0.14	0.14	0.14
Natural antioxidant (dry)	0.04	0.04	0.04
Chicken fat + antioxidant^†^	5.0	5.0	5.0
Flavor powder^†^	1.0	1.0	1.0
Nutrient, Dry Matter Basis (DMB) %	CGM	SBM	CFP
Moisture	5.56	7.02	3.74
Energy (kcal/kg)^**^	3,260	3,117	3,261
Crude protein	39.9	36.2	39.2
Crude fat	12.4	12.5	15.3
Total dietary fiber	12.9	14.8	18.0
Ash	6.18	7.29	7.07
Total starch	33.0	28.8	25.7
Gelatinized starch	29.8	25.4	23.6

^*^Corn gluten meal (CGM), soybean meal (SBM), and corn-fermented protein (CFP).

^†^Indicates ingredient was applied topically after extrusion and drying.

^**^Calculated metabolizable energy.

### Diet Production

Diets were mixed as three separate batches and produced over three replicate processing days. After mixing, diets were added to an overhead bin with live bottom feeder which conveyed the mix to the preconditioner at an average feed rate of 285.76 kg/h. In the preconditioner, moisture and thermal energy were added in the form of water and steam to begin the process of hydration and starch gelatinization, respectively. Water and steam inputs were recorded approximately every 20 min. Preconditioner paddles rotated at a speed of 165 rpm to effectively mix the matrix. Material exited the preconditioner into the extruder at a temperature of 85 °C and an average total mass flow (TMF) of 335 kg/h.

A small production-scale single-screw extruder (model E525; Extru-Tech, Sabetha, KS) was used for this experiment. The following processing parameters were recorded every 20 min: injection of water in kg/h, extruder rotations per minute (RPM), die temperature, die pressure, percent openness of the mass restriction valve (MRV), bulk density out of the extruder (g/L), and percent load, which was calculated as the actual load in amps divided by the maximum extruder load (186 A). Material flowed through the extruder at an average TMF of 345 kg/h. These data were used to calculate the specific mechanical energy (SME) using the following equation (equation 1).


$$\begin{array}{l} SME\;\left(\frac{kJ}{kg}\right)=\;\frac{\frac{\tau -\tau_{o}}{100}*\left(\frac{N}{N_{r}}\right)*P_{r}}{m} \end{array}$$ (1)

where τ is the percent torque, or motor load, τ_o_ is the no-load torque (18.71%), *N* is the screw speed in rpm, *N*_r_ is the rated screw speed (425 rpm), *P*_r_ is the rated motor power (111.85 kW), and *m* is the TMF in kg/s. The in-barrel moisture content (IBM) was also calculated using the equation below (equation 2).


$$\begin{array}{l} IBM=\frac{m_{f}\times X_{f}+m_{ps}+m_{pw}+m_{es}+m_{ew}}{m_{f}+m_{ps}+m_{pw}+m_{es}+m_{ew}} \end{array}$$ (2)

where *m*_f_ is the feed rate, *X*_f_ is the moisture content of the raw material, *m*_ps_ is the percentage of added steam in the preconditioner, *m*_pw_ is the percentage of added water in the preconditioner, *m*_es_ is the percentage of steam added into the extruder, and *m*_ew_ is the percentage of water added into the extruder. A moisture content of 10% was assumed for *X*_f_. A 3.2 mm die was used for all diets to produce the food in an appropriate size for both dogs and cats. Knife speed was kept constant at 1,300 rpm. The MRV, which is located directly behind the die plate on the extruder, was used to aid in controlling the flow of material through the extruder by either increasing or decreasing constriction. This valve was utilized to aid in expansion of kibbles in order to achieve a similar bulk density out of the extruder, which was used as a reference point for product consistency.

Kibbles were dried on aerated cookie sheets in a forced air convection oven at approximately 141 °C. In-process product moisture was determined by IR heat lamp (DSH-50-1; WANT Balance Instrument Co., Ltd, Changzhou, China) and kibbles were considered dry when moisture was less than 10%. Kibbles were separated into aliquots for dogs and cats and then coated with chicken fat (5.0%) fortified with antioxidant preservatives (0.03%), and species-appropriate powdered flavor (1.0%). Coated diets were stored in 9 kg poly-lined Kraft paper bags until fed. Within the bags replicates were composited.

### Kibble Analysis

Ten kibbles were collected for measurement at 20-min intervals during each extrusion replicate. Digital calipers were used to measure the length and diameter of kibbles. The recorded diameter was an average of two diameter measurements taken by rotating the piece 90°, as kibbles were not uniformly symmetrical. Kibble weight was also recorded using an analytical scale with 0.1 mg sensitivity (EX324N; OHAUS Corporation, Parsippany, NJ). This information was used to calculate the sectional expansion index (SEI; mm^2^/mm^2^), specific length (*l*_sp_; mm/g) to assess radial and longitudinal expansion, as well as the volume (*V*_e_; cm^3^) and the piece density (ρ; g/cm^3^), using the equations described below:


$$\begin{array}{l} SEI=\frac{D_{e}^{2}}{D_{d}^{2}} \end{array}$$ (3)

$$\begin{array}{l} l_{sp}=\frac{l_{e}}{m_{e}} \end{array}$$ (4)

$$\begin{array}{l} V_{e}=\frac{\frac{\pi }{4}*D_{e}^{2}*\;l_{e}}{1,000} \end{array}$$ (5)

$$\begin{array}{l} \rho =\frac{m_{e}}{V_{e}} \end{array}$$ (6)

where *D*_e_ is the diameter of the extrudate in mm, *D*_d_ is the diameter of the die used (3.2 mm), *l*_e_ is the length of the extrudate in mm, and *m*_e_ is the mass of the extrudate in g.

Kibbles were analyzed for hardness and toughness using a texture analyzer (TA-XT2 Texture Technologies Corporation, Hamilton, MA). The procedure used was modified from Dogan and Kokini (2007), wherein a total of 20 kibbles per collection point per day were measured, amounting to 180 kibbles total per treatment. A 25-mm cylindrical probe was used for a compression test with a pretest speed of 2 mm/s, a test speed of 1 mm/s, and a posttest speed of 10 mm/s. The strain level for the test was 50%. Hardness was considered to be the peak fracture force, or the maximum force at which a fracture occurs in each compression signature and was measured in kg. The toughness was considered to be the energy required to completely disintegrate the sample and was calculated as the total area under the curve in each compression signature in kg × mm.

### Feeding Trial

Twelve intact beagles (8 male, 4 female) with average weight of 10.99 ± 1.24 kg were used for the feeding study. To be included in the study, the dogs were required to be in good health, with no preexisting conditions. The dogs were individually housed in 1.83 m × 1.20 m cages with acrylic-coated mesh flooring with a three-piece tray underneath to allow for separation of urine and feces. The study consisted of three periods comprised of 9 d of diet adaptation, followed by 5 d of collection in a replicated Latin Square (3 × 3) experimental design, according to the procedure defined by Kim et al. (2009). Wherein each treatment was fed in each period over the three periods (6 wk total). In this model, each animal served as its own control, and each treatment had 12 total observations. Dogs were housed at the Large Animal Research Center at Kansas State University in Manhattan, KS.

Animals were housed at 22 °C and 50% relative humidity (RH), and water was provided ad libitum. Lights were on a 12-h cycle with lights off from 1900 to 0700 each night. The beginning food amounts were estimated to maintain body weight using the NRC (2006) equations to estimate the metabolizable energy (ME) of the food and food amounts as 130 × body weight (BW)^0.75^ for dogs. Dogs were weighed after each period and feeding amounts were adjusted accordingly. Feeding occurred twice daily at 0800 and 1600. Animal caretakers were not blinded to the dietary treatments, as they were required to weigh out each meal to meet each animal’s specific caloric needs. Food was offered for 1 h at each meal, and remaining orts were then collected and weighed.

Following the 9 d of adaptation, sample collection began starting at 0800 and extended for the next 120 h. Feces were collected after meals and whenever observed throughout the period. Fecal samples were scored on a 5-point scale created by Royal Canin: (1) completely liquid stool that can be poured, (2) very soft stool that takes the shape of its container, (3) soft stool that retains shape, (4) hard formed stool, and (5) hard dry pellets (Royal Canin, St. Charles, MO). Samples were scored in 0.5 increments, and a score of 3.5 to 4 was considered ideal. Samples were placed in plastic bags (Whirl-pak, The Aristotle Corporation, Stamford, CT) labeled with dog name, period number, and day of study. Samples from each period were pooled and frozen for later analysis.

### Digestibility Calculations

At the conclusion of the feeding assay, all feces were dried until no additional weight was lost (24 to 48 h) in an electric oven (Cat 52755-20, Matheson Scientific, Morris Plains, NJ) at 55 °C. Dried samples were ground using a high-speed fixed blade rotor mill to pass through a 1-mm screen (ZM 200, Retsch, Verder Scientific, Haan, Germany). Titanium (Ti) concentration was measured in food and feces by use of a microplate reader (Synergy H1, Biotek, Winooski, VT), as described by Myers et al. (2004). ATTD by TiO_2_ was determined by the following equation:


$$\begin{array}{l} Nutrient\;digestibility=\left[1-\frac{\left(\%\;TiO_{2}\;in\;food*\%\;nutrient\;in\;feces\right)}{\left(\%\;TiO_{2}\;in\;feces*\%\;nutrient\;in\;food\right)}\right]*100\; \end{array}$$ (7)

### Nutrient Analysis

Experimental ingredient, feed, and fecal samples were analyzed for nutrient composition at a commercial laboratory (Midwest Laboratories, Omaha, NE). Analysis included moisture and dry matter (DM; AOAC 930.15), organic matter (OM; AOAC 942.05), crude protein (CP; AOAC 990.03), and fat by acid hydrolysis (AOAC 954.02). Total starch and gelatinized starch were analyzed at Wenger Technical Center (Sabetha, KS; Mason and Gleason, 1982). Analysis for total dietary fiber (AOAC 985.29) was performed at Kansas State University. Gross energy was measured using bomb calorimetry according to the methods defined in the Parr operating manual (1341 Oxygen Bomb Calorimeter, Parr Instrument Company, Moline, IL). Nutrient analysis of experimental ingredients and diets are presented in Tables 1 and 2.

### Palatability Trial

Experimental treatments were evaluated for palatability by both dog and cat panels at a commercial kennel (Summit Ridge Farms, Susquehanna, PA). Each was conducted as a split-plate test, in which two stainless steel bowls each containing 400 g of food for dogs, and 100 g of food for cats were presented to animals for a total of 30 min before removal. Bowl positions were switched daily. Twenty animals were fed each day of the study, and each comparison trial was repeated for 2 d, providing a total of 40 observations for each species and paired comparison test. Preference was observed by the technicians who recorded the animals first choice when approaching the food bowls, and total food consumption. Data from consumption are presented as a ratio (equation 8).


$$\begin{array}{l} Intake\;Ratio\;(IR)=\left(\frac{consumption\;of\;diet\;A}{total\;consumption\;of\;diet\;A+diet\;B}\right) \end{array}$$ (8)

### Statistical Analysis

All statistical analysis was conducted with Statistical Analysis Software (SAS version 9.4; SAS Institute, Inc., Cary, NC). The averages in both the extrusion and digestibility studies were analyzed using generalized linear mixed models (proc GLIMMIX). The model statement for extrusion parameters contained preconditioner added water, preconditioner added steam, extruder added water, extruder rpm, die temperature, die pressure, percent openness of the MRV, percent load, SME, and bulk density as fixed variables. The model statement for kibble measurements contained length, diameter, weight, density, SEI, and specific length as fixed variables. The model statement for the texture analysis contained hardness and toughness as fixed variables. All model statements included production day as a random variable, and all means were separated using Fisher’s LSD with a significant *F* (α = 0.05). The model statement for digestibility contained diet as a fixed variable, and period and dog were included as random variables. Means were separated using Fisher’s least significant difference (LSD) with a significant *F* (α = 0.05). In the palatability experiments, the consumption ratio was analyzed using a two-way analysis of variance (ANOVA), and the first-choice preference was analyzed using a chi-square test.

## RESULTS

### Processing Parameters

The CFP diet had more (37.93 kg/h; *P* < 0.05) water injected into the preconditioner than the CGM diet (37.82 kg/h), but SBM (37.89 kg/h; Table 3) was similar (*P* > 0.05) to both. Steam addition into the preconditioner was similar among dietary treatments (average 53.46 kg/h; *P* > 0.05). Likewise, water added to the matrix in the extruder (average 10.91%), extruder RPM (average 224.25 rpm), TMF (average 343.86 kg/h), die temperature (average 103.17 °C), percent load (average 36.00%), SME (average 109.69 kJ/kg), and IBM (average 19.62%) were also similar among treatments (*P* > 0.05; Table 3). Die pressure was highest (2,988 kPa; *P* < 0.05) during the extrusion of the CFP diet, followed by the SBM diet (2,528 kPa), with the least amount of pressure recorded for the CGM diet (1,666 kPa; Table 3). On the first day (replicate 1) of extrusion for the CFP diet and the second day (replicate 2) of extrusion for the SBM diet, it was discovered that one of the die openings had become blocked, resulting in increased pressure in the barrel. Moreover, the treatments also differed (*P* < 0.05) in the percent openness of the MRV. The CGM diet had the largest opening at 60.00%, followed by SBM at 51.67% and CFP at 40.00% (Table 3). A similar bulk density (out of the extruder [OE]) was achieved (average 362.9 g/L; *P* > 0.05).

**Table 3.** Processing parameters recorded in preconditioner and extruder during diet production

		Diet^*^				
	Item	CGM	SBM	CFP	SEM	*P*-value
Preconditioner	Water, kg/h	37.82^b^	37.89^ab^	37.93^a^	0.08	0.0375
	Steam, kg/h	53.92	53.20	53.25	0.54	0.6223
Extruder^**^	Water, kg/h	10.90	11.32	10.51	0.68	0.5031
	RPM	245.0	225.8	209.2	33.19	0.7616
	TMF, kg/h	345.5	345.1	345.1	0.55	0.5744
	Die temperature, °C	104.2	105.6	106.2	3.08	0.4598
	Die pressure, kPa	1,666^c^	2,528^b^	2,988^a^	81.25	0.0008
	MRV, % open	60.00^a^	51.67^b^	40.00^c^	0.96	<0.0001
	Percent load	36.55	35.92	35.55	1.45	0.4669
	SME, kJ/kg	120.3	109.7	91.65	22.18	0.6144
	Bulk density, g/L	354.9	367.8	366.1	5.94	0.2811
	IBM, %	20.02	19.38	19.46	0.42	0.2768

^*^Corn gluten meal (CGM), soybean meal (SBM), and corn-fermented protein (CFP).

^**^Rotations per minute (RPM), total mass flow (TMF), mass restriction valve (MRV), specific mechanical energy (SME), in-barrel moisture content (IBM).

^a–c^Indicates that within a row, unlike letters differ (*P* < 0.05).

### Kibble Measurements

The length of kibbles was similar among treatments, averaging 7.49 mm (*P* > 0.05; Table 4). Conversely, the kibble diameter was larger (*P* < 0.05) when CGM and SBM were added to the diet (5.66 and 5.60 mm, respectively) compared to the diet containing CFP (5.18 mm; Table 4). Kibble mass (average 0.0899 g), volume (average 0.1772 cm^3^), and piece density (average 0.5112 g/cm^3^; Table 4) were similar (*P* > 0.05) among treatments. The similarities among treatments in kibble length and mass are reflected in the calculated specific length, which indicated no difference (*P* > 0.05) between treatments in longitudinal expansion (average 81.16 mm/g). Additionally, the differences (*P* < 0.05) in diameter are mirrored in the SEI, with CGM and SBM diets having a larger kibble expansion index (3.13 and 3.07 mm^2^/mm^2^, respectively) compared to CFP kibbles (2.62 mm^2^/mm^2^; Table 4).

**Table 4.** Kibble measurements and calculations

	Diet^*^				
Item^**^	CGM	SBM	CFP	SEM	*P*-value
Length, mm	6.66	8.11	7.69	0.73	0.4337
Diameter, mm	5.66^a^	5.60^a^	5.18^b^	0.05	0.0040
SEI, mm^2^/mm^2^	3.13^a^	3.07^a^	2.62^b^	0.23	0.0046
Specific length, mm/g	84.83	81.35	77.29	4.29	0.4910
Mass, g	0.0793	0.1007	0.0897	0.01	0.4093
Volume, cm^3^	0.1682	0.2007	0.1626	0.02	0.2839
Piece density, g/cm^3^	0.4760	0.5039	0.5537	0.01	0.4327
Hardness, kg	3.79	4.53	4.80	0.32	0.1411
Toughness, kg × mm	3,223	2,981	2,666	781.1	0.8834

^*^Corn gluten meal (CGM), soybean meal (SBM), and corn-fermented protein (CFP).

^**^Sectional expansion index (SEI).

^a–c^Indicates that within a row, unlike letters differ (*P* < 0.05).

Analysis of kibble texture revealed no differences among hardness and toughness among all treatments (*P* > 0.05, Table 4). The treatments averaged a hardness of 4.37 kg and an average toughness of 2,956.98 kg × mm.

### Feeding Trial

Results for food intake, fecal output, fecal score, and fecal weight are presented in Table 5. There was no difference (*P* > 0.05) in food intake among all diets. This was expected as food intake was controlled to maintain body weight and fed as a meal twice daily. Dogs were observed to be eager to consume food, and any orts recorded were generally the result of spilled food from a respective dog. Dogs fed the CGM diet had fewer daily defecations (*P* < 0.05) than those fed the SBM or CFP diets (2.03 vs. average 2.41, respectively). Fecal output (dry) was lowest (*P* < 0.05) for dogs fed the CGM (35.91 g/d) compared to both other treatments, with 20% greater fecal output for those fed SBM and nearly 55% greater fecal output for dogs fed the CFP diet, respectfully. The dogs consuming CGM had a lower (3.27; *P* < 0.05) fecal score to those fed the CFP diet (3.63), with dogs fed the SBM diet being intermediate (3.43; Table 5).

**Table 5.** The effect of experimental diets on food intake, daily defecations, fecal score, and dry fecal weight

	Diet^*^				
Item	CGM	SBM	CFP	SEM	*P*-value
Food intake, g/d	221.06	223.80	225.68	4.31	0.5130
Daily defecations	2.03^b^	2.43^a^	2.38^a^	0.13	0.0124
Wet fecal weight, g/d	108.31^b^	151.36^a^	154.29^a^	4.46	<0.0001
Dry fecal weight, g/d	35.91^c^	43.25^b^	55.65^a^	7.17	<0.0001
Fecal score^†^	3.27^b^	3.43^ab^	3.63^a^	0.09	0.0074

^*^Corn gluten meal (CGM), soybean meal (SBM), and corn-fermented protein (CFP).

^†^Samples were scored on a 5-point scale: (1) completely liquid stool that can be poured, (2) very soft stool that takes the shape of its container, (3) soft stool that retains shape, (4) hard formed stool, and (5) hard dry pellets.

^a–c^Means within a row with unlike letters differ (*P* < 0.05).

### Apparent Total Tract Digestibility

The DM digestibility was lower (*P* < 0.05) for dogs fed the CFP diet vs. both CGM and SBM diets (78.19% vs. 83.37% and 80.61%, respectively; Table 6). OM digestibility was also lower for CFP diets (*P* < 0.05; 79.46%) relative to dogs fed CGM and SBM (86.17% and 83.13%, respectively). CP digestibility did not vary between SBM and CFP (average 82.40%; *P* > 0.05), but both were less digestible than CGM (*P* < 0.05; 85.65%). Crude fat digestibility was lower (*P* < 0.05) for dogs fed the CFP diet vs. both CGM and SBM diets (90.16% vs. average 91.57%). Dogs fed the CGM diet had the highest (*P* < 0.05) digestibility of dietary fiber (57.39%) when compared to the SBM and CFP diets, which did not differ from each other (average 47.71%). Ash digestibility was higher (*P* < 0.05) for dogs fed the SBM diet than for dogs fed the CGM diet (39.57% vs. 31.57%, respectively), with CFP similar (*P* > 0.05) to both (35.61%). The dogs fed the CGM also had the highest (*P* < 0.05) digestibility of gross energy (77.16%) when compared to both SBM and CFP diets (75.48% and 69.89%, respectively).

**Table 6.** The effect of experimental diets on apparent total tract digestibility as determined by titanium concentration

	Diet^*^				
Item, %	CGM	SBM	CFP	SEM	*P*-value
Dry matter	83.37^a^	80.61^b^	78.19^c^	0.36	<0.0001
Organic matter	86.17^a^	83.13^b^	79.46^c^	0.38	<0.0001
Crude protein	85.65^a^	82.59^b^	82.21^b^	0.45	<0.0001
Crude fat	91.41^a^	91.72^a^	90.16^b^	0.25	<0.0001
Total dietary fiber	57.39^a^	49.98^b^	45.44^b^	2.16	0.0001
Ash	31.57^b^	39.57^a^	35.61^ab^	1.79	0.0150
Gross energy	77.16^a^	75.48^b^	69.89^c^	0.63	<0.0001

^*^Corn gluten meal (CGM), soybean meal (SBM), and corn-fermented protein (CFP).

^a–c^Means within a row with unlike letters differ (*P* < 0.05).

### Palatability Trial

On a consumption basis, the dogs preferred (*P* < 0.05) CGM over CFP, roughly 2:1 (Table 7). There was no difference (*P* > 0.05) between the CGM and SBM (IR of 0.432), or between SBM and CFP (IR of 0.454). When evaluating which food was approached first by the dogs, there was no difference (*P* > 0.05) between CFP and CGM (17 vs. 23) or between SBM and DDG (20 vs. 20). However, there was a difference (*P* < 0.05) between SBM and CGM, with 13 observations of an approach to the SBM first and 27 observations of an approach to the CGM first over the 2-d trial. The cats displayed different preferences to the diets than dogs (Table 7). In this case CFP and SBM were preferred (*P* < 0.05) over the CGM diet with an IR of 0.606 and 0.632, respectively. There was no difference between SBM and CFP (IR of 0.456; *P* > 0.05). No difference (*P* > 0.05) was seen in first approach between any of the paired comparisons over the 2-d trial.

**Table 7.** The effect of experimental diets on palatability assessed by dogs and cats

	Dog		Cat	
Diet^*^ comparison, A vs. B	FC^†^	IR of diet A^‡^	FC^†^	IR of diet A^‡^
CFP vs. CGM	17	0.365^**^	22	0.606^**^
SBM vs. CGM	13^**^	0.432	19	0.632^**^
SBM vs. CFP	20	0.454	20	0.456

^*^Corn gluten meal (CGM), soybean meal (SBM), and corn-fermented protein (CFP).

^†^First choice (FC): number of first visits to bowl A (out of a total of 40 observations).

^‡^IR of diet A = intake (g) of diet A/total intake (g) of diets A + B.

^**^Comparison differs *P* < 0.05.

## DISCUSSION

### Diet Formulation

Because the primary goal of this work was to examine the use of CFP as a potential ingredient for commercial pet foods, CGM and SBM were chosen for comparison due to their current use in the pet food industry, and their comparable protein levels. However, inclusion of traditional DDGS in this study may have been beneficial as this ingredient is similar to and has been researched more than CFP. Because these ingredients are produced from the same processes and are nutritionally similar, with the exception of protein content, previous work examining the use of DDGS may also be representative of CFP. Future studies should be conducted to directly compare these ingredients and their use in pet foods.

The experimental diets were developed on a platform of 25% inclusion of the CFP with corresponding quantities of protein from the CGM and SBM. During their development standard CP values for ingredients (CGM: 60%, SBM: 49.6%, CFP: 49.2%) were used with an initial estimate of CP between 33% and 34%. However, the CP content of each of the individual ingredients and ration were greater than initially assumed with a final diet CP analysis that was higher than expected. Likewise, the crude fat values did not meet the expected value of 12% to 13% among the diets. This was in part controlled by the external application of fat during the coating process. But, as a raw material the CFP had more than double the fat content of the CGM and SBM, resulting in 3% more crude fat in the CFP diet. Otherwise, the experimental diets followed the initial experimental design and were produced in a fashion to yield similar products from a visual and physical perspective conducive to the study.

### Processing Parameters

The unique composition of each of the proteins evaluated in this study, especially in regard to their starch and fiber contents, resulted in various processing changes required during extrusion in order to produce a similar product. During production of the CFP ingredient, a large portion of the starch content is removed in the fermentation process. As a result, this ingredient is less prone to expansion during extrusion, and required some changes in processing parameters to produce a similar product (Chevanan et al., 2007; Stein and Shurson, 2009). Reported starch concentrations for traditional DDGS have varied, with values ranging from 1.9% to 8.2% (Buenavista et al., 2021). The total starch for the CFP was in the lower end of this range, at 2.73%. During extrusion, high moisture and temperatures added in the preconditioner aid in the gelatinization of starch (Tran et al., 2008). This may be why CFP matrix required slightly more water in the preconditioning step, in order to further promote the gelatinization of the remaining starch. Conversely, CGM, which originates from the same cereal as CFP (corn), is well known for its high starch content. This starch readily gelatinizes, which is likely why this diet needed less hydration in the preconditioning step (Belyea et al., 2004).

In addition to moisture and heat, increased mechanical energy can also aid in the gelatinization of starch. By closing the MRV diameter, more back-pressure is created which results in more friction behind the die plate, and this reduced flow in the extruder barrel translates to more mechanical energy. This increase in friction helps to further gelatinize the starch (Riaz, 2000). This is further supported by the differences seen between the treatments in die pressure, in which the CFP diet required more die pressure than the CGM and SBM diets to achieve a similar bulk density. As material exits the extruder die, the drastic change in pressure causes the water trapped in the gelatinized starch matrix to vaporize, leaving an empty cell, and resulting in an expanded and puffy product (Kannadhason et al., 2010). By increasing the pressure behind the die, this change in pressure as the extrudate exits the barrel is increased, resulting in a lighter density. High lipid levels are counterproductive to pressure buildup within the extruder barrel due to lubrication effects which in turn reduce the application of mechanical energy and product expansion (Riaz and Rokey, 2012). The CFP had the highest lipid content among the treatments, which may be another reason why this treatment required a smaller diameter MRV opening to increase pressure at the die. By manipulating pressure within the barrel, it is possible to control the expansion of the extrudate, and ultimately create comparable products even with varying starch levels. This is reflected in the analyzed starch values of the experimental diets, which are similar when comparing the values of the percent of starch cook (88% to 92% of total starch).

### Kibble Measurements

Despite having similar bulk densities out of the extruder, there were differences between treatments in both the diameter and radial expansion of the kibbles. A study by Hsieh et al. (1989, 1991) found that increasing fiber content can decrease radial expansion. The CFP had the highest fiber content among treatments, which was likely responsible for smaller SEI. Various studies examining the use of DDGS, which are similar to CFP in their high-fiber composition, found that the addition of DDGS to extruded aquaculture feed decreased the expansion ratio of the feed when compared to a control diet without DDGS (Chevanan et al., 2004; Kannadhason et al., 2010). Despite this, there were no differences in the mass, volume, or piece density of the kibbles. The ability to maintain similarities in piece density between treatments was achieved and further supports the conclusion that it is possible to create similar products with the treatments used with minimal changes to processing parameters.

### Feeding Study

Higher levels of total dietary fiber in the CFP and CBM diets are likely responsible for the differences seen in daily defecations, fecal mass, and fecal score when compared to the CGM diet. High levels of fiber may increase the rate of passage through the digestive system and decrease absorption, resulting in less overall digestion, and larger fecal mass (Allen et al., 1981; Yamka et al., 2003). The fecal scores, while differing between treatments, were all within the acceptable range, e.g., 3.0 to 4.0. Fecal scores for SBM diets in this study were similar to those recorded by Bednar et al. (2000) and Clapper et al. (2001). Clapper et al. (2001) also explored levels of insoluble vs. soluble fiber in SBM and reported that SBM had nearly a 10:1 ratio of insoluble to soluble fiber. Insoluble fiber can aid in the formation of ideal, firm feces (Burkhalter et al., 2001). This is an area that should be explored more fully in the current work. Additionally, higher fiber levels can be supplemented in diets to aid in body weight control and promote regular defecation (de Godoy et al., 2009), suggesting possible benefits for these ingredients beyond simply providing protein.

The effect of the higher levels of total dietary fiber was also reflected in the DM and OM digestibility of the diets. Both DM and OM digestibility were higher when the dogs were fed lower fiber diets. These results are corroborated by the findings of previous studies (Zuo et al., 1996; Bednar et al., 2000; Carciofi et al., 2009), wherein a DM digestibility of 81% was reported for dogs fed a CGM diet, and an average DM digestibility of 78.3% was reported in dogs fed an SBM-based diet. Similarly, a study by Risolia et al. (2019) found a comparable DM digestibility for dogs fed a diet with DDGS, reporting an ATTD of 76.8% when DDGS were included at 20%. The digestibility values for both DM and OM met our expectations and, while differing between treatments, would all be considered acceptable for a commercial pet food.

CP digestibility values observed in this study met expectations and were consistent with previous literature. The results agree with the data reported by Carciofi et al. (2009) in which they found cats fed a diet including 17.2% CGM had a CP digestibility of 84%. While a different species, these animals share enough similarities to extrapolate results from this type of evaluation. The digestibility of crude fat was slightly less for those fed the CFP diet; however, numerically the treatments were quite similar, and the reported value is analogous to previously observed values (Silva et al., 2016). Again, while the experimental diets differed slightly in digestibility, all would be considered acceptable.

One could concede that the level of the three protein sources was higher than typically considered practical. Further, previous research has shown detrimental effects on animal utilization due to elevated levels of oligosaccharides from the SBM at 30% inclusion as an example. Oligosaccharides are nonstarch polysaccharides (NSPs) that are highly fermentable in the large intestine, and an excess can result in decreased digestibility, increased flatulence, and (or) loose stools (Félix et al., 2012). These high levels of NSP are present not only in SBM, but in derivatives of DDGS as well. An increased presence of NSP could explain the decrease in gross energy digestibility in both SBM and CFP treatments, as the added fibers could act as a caloric diluent. Both Félix et al. (2012) and Silva et al. (2016) have examined the use of similar ingredients at graded levels in dog foods along with the addition of enzyme complexes to aid in digestion to diminish the effect of NSPs. Both studies reported increased digestibility by the dogs fed foods with added enzymes, and a decrease in digestibility with increasing levels of added protein sources. Different from these previous studies, the elevated level chosen for the current research was intended to test the ingredients as a major contributor of protein in the diet and to increase the chances of identifying sensory differences. In this case, the CFP proved to be slightly less digestible than CGM and equal to SBM. No overt sign of negative effects on stools or digestibility were observed. Thus, it appears the elevated level included could be acceptable in the face of high-protein diets (exceeding requirements by 25%) and (or) for production of feline diets that typically exceed 30% CP.

### Palatability

Because the CFP diet had the highest fat content, it was predicted that this diet would be favored by the dogs. Dogs have a high preference for fats (Li et al., 2017) and a dog’s food selection is highly driven by smell. Houpt et al. (1978) found that when dogs were presented with a bland diet supplemented with a meat odor, they preferred it over a control diet with no odor. Oils and short-chain fatty acids are recognized in a special section of the olfactory bulb, and this strong odor may be a driving factor behind a dog’s liking (Manabe et al., 2010). Despite this, the CFP diet was not preferred by the dogs. These preferences are supported by a study conducted by Li et al. (2017), who found that dogs have a liking for corn starch over other plant-based starch sources. While CGM and CFP are both derived from corn, the addition of the corn starch to the CGM diet could have driven the liking of this food. One might consider rice as the starch source in a control for future work so as to not confound the evaluation of the proteins and serve as a blander base ingredient to evaluate the various corn and soy-derived protein concentrates.

The cats displayed different preferences to the diets than dogs. The aversion to the CGM diet was unexpected, as cats have a high preference for amino acids (White and Boudreau, 1975). Given the CGM diet was almost 4 percentage units higher in protein relative to the SBM diet, and slightly higher than the CFP diet it would have been hypothesized to be preferred. However, the higher affinity to the CFP diet for cats, in comparison to preferences shown by dogs, may be due to the high levels of yeast present in CFP. As DDGS are a product of fermentation, much of their protein content can be attributed to yeast (Belyea et al., 2004). Because CFP is produced from these same processes, it can be inferred that a large portion of its protein content can also be ascribed to yeast. Yeast has been observed as highly palatable to cats, most likely due to the high presence of nucleotides (White and Boudreau, 1975; Swanson and Fahey, 2004). More studies should be done with both species to evaluate palatability of CFP to confirm these initial results. In spite of this, the quantity of each should have been effective to determine if any unwanted sensory attributes were present in the protein sources. The results support they were well-liked and effective for use in pet foods.

## CONCLUSION

As it relates to extruded pet food, the use of CFP in exchange for SBM and CGM can be managed to produce a kibble of similar size, shape, and density appropriate for the pet food market. The dogs fed CFP had high-quality stools that were higher in mass than dogs fed both SBM and CGM. Digestibility assessment in dogs indicated that the CFP diet was overall less digestible than SBM and CGM diets but had similar protein digestibility compared to the SBM diet. However, when used in a well-balanced diet, all ingredients would be viable options for pet foods. Both dogs and cats found the food to be palatable and showed no signs of refusal. Future work should explore practical inclusion levels of CFP and the resulting impact on digestibility, as well as the value derived from the yeast component on extra-nutritional value and their influence on palatability.
